# Separation of Folinic Acid Diastereomers in Capillary Electrophoresis Using a New Cationic *β*-Cyclodextrin Derivative

**DOI:** 10.1371/journal.pone.0120216

**Published:** 2015-03-17

**Authors:** Jia Yu, Xinlei Liang, Zhaokun Wang, Xin Guo, Tiemin Sun, Xingjie Guo

**Affiliations:** 1 Department of Pharmaceutical Analysis, School of Pharmacy, Shenyang Pharmaceutical University, Ministry of Education, Shenyang, Liaoning Province, P. R. China; 2 Key Laboratory of Structure-Based Drug Design and Discovery, Shenyang Pharmaceutical University, Ministry of Education, Shenyang, Liaoning Province, P. R. China; Tecnologico de Monterrey, MEXICO

## Abstract

A method for the separation of folinic acid diastereomers by capillary electrophoresis in chiral separation media was developed. Aiming to achieve a good separation of the anionic analytes, a newly synthesized cationic *β*-cyclodextrin derivative, mono-6-deoxy-6-piperdine-*β*-cyclodextrin, was applied as the chiral selector. The effect of background electrolyte pH, the concentration of the cyclodextrin additive, and organic modifier on the separation was investigated. A good separation of folinic acid diastereomers was obtained with 30 mmol/L phosphate buffer at pH 6.50 containing 6.0 mmol/L of mono-6-deoxy-6-piperdine-*β*-cyclodextrin in 10% acetonitrile. Based on the capillary electrophoresis data, the binding constants of each diastereomer with mono-6-deoxy-6-piperdine-*β*-cyclodextrin were determined. Moreover, a computational modeling study, using the semi-empirical PM3 method, was used to discuss the possible mechanism of separation of folinic acid with mono-6-deoxy-6-piperdine-*β*-cyclodextrin.

## Introduction

Capillary electrophoresis (CE) has developed within the last decade into a powerful analytical tool for the separation of drug stereoisomers [1, 2]. Despite the fact that the most important stereoisomer separations are separations of enantiomers, utilization of CE for separations of diastereomers is also challenging and attracting more interests, especially in chiral separation media [3]. Therefore, addition of proper chiral selectors to the background electrolyte (BGE) can be used as a means of achieving satisfactory diastereomer separation [4]. Cyclodextrin (CD) and its derivatives are the most frequently used chiral selectors in CE. In recent years, charged CD derivatives as chiral selectors have been extensively used in CE for the separation of many enantiomers as well as of diastereomers [5, 6]. However, most of the used CDs are mixtures with varying degrees and location of substitution. In the case of these randomly multisubstituted derivatives, the reproducibility in separation and synthesis cannot always be guaranteed and the discussion of possible separation mechanisms is difficult. Thus, single-isomer CD derivatives, have gained more and more interests and been recommended extensively [7].

**Fig 1:**
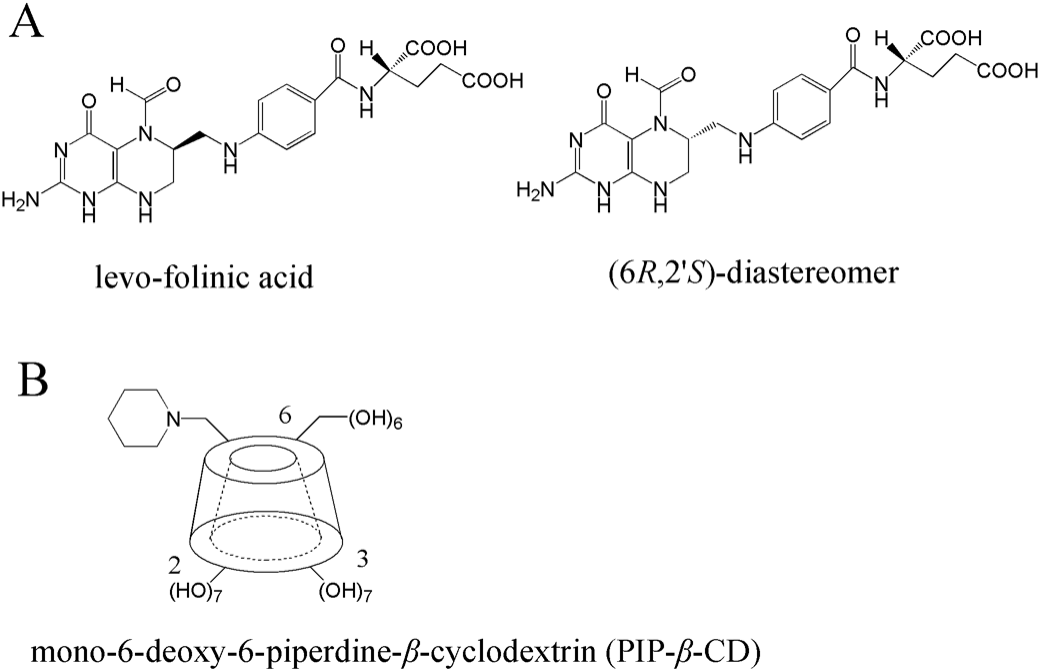
The chemical structures of (A) folinic acid diastereomers and (B) mono-6-deoxy-6-piperdine-*β*-cyclodextrin (PIP-*β*-CD).

Folinic acid, generally administered as calcium or sodium folinate, is an adjuvant used in cancer chemotherapy involving the drug methotrexate [8]. Folinic acid contains 2 centers of asymmetry. A large majority of the commercial pharmaceutical preparations used in therapy contain a pair of diastereomers, a 1:1 mixture of levo-isomer (6*S*,2'*S*-configuration) and dextro-isomer (6*R*,2'*S*-configuration) (Fig. 1A). The levo-isomer or levo-folinic acid, is the only pharmacologically active optical isomer. Preclinical studies have shown that the dextro-isomer of folinic acid, (6*R*,2'*S*)-diastereomer, having no biological activity might compete with the levo-folinic acid for transport into cells [9]. Therefore, a proper method to separate folinic acid diastereomers is very necessary and meaningful.

Taking into account the successful use of charged *β*-cyclodextrin derivatives to separate the opposite charged analyte stereoisomers, the application of cationic *β*-cyclodextrin derivatives for folinic acid diastereomers should be worth studying. Therefore, in our study, a new cationic single-isomer CD derivative, mono-6-deoxy-6-piperdine-*β*-cyclodextrin (PIP-*β*-CD) [10] (Fig. 1B), was synthesized and utilized as the selector in the BGE for the separation of diastereomers of folinic acid by CE. The resolution was optimized by examining the influence of some experimental variables such as BGE pH, organic modifier and CD concentration. Based on the CE data, the binding constants were also determined, which was helpful to confirm and predict the optimum CD concentration. Furthermore, to have insight into the mechanism of separation of folinic acid by PIP-*β*-CD and to rationalize our experimental results, the semiempirical method PM6 [11–13] was employed to understand the inclusion conformation and the molecular electrostatic interaction between folinic acid and PIP-*β*-CD.

## Materials and Methods

### Materials

Calcium folinate was purchased from the National Institute for the Control of Pharmaceutical and Biological Products (Beijing, China). Levo-folinic acid was kindly provided by the Pharmaceutical Chemistry Laboratory of Shenyang Pharmaceutical University. *β*-Cyclodextrin (*β*-CD) was obtained from Tianjin Bodi Chemical Holding Co., Ltd. (Tianjin, China). Carboxymethyl-*β*-cyclodextrin (CM-*β*-CD) and hydroxypropyl-*β*-cyclodextrin (HP-*β*-CD) were obtained from Shandong Binzhou Zhiyuan Bio-Technology Co., Ltd. (Shandong, China). Sulfated-*β*-cyclodextrin S-*β*-CD (S-*β*-CD) was purchased from Sigma-Aldrich (St.Louis, MO, USA). Sodium dihydrogen phosphate (NaH_2_PO_4_) and phosphoric acid (H_3_PO_4_) were of analytical grade and obtained from Shantou Xilong Chemical Co. Ltd. (Shantou, China). Double distilled water was used throughout the study.

### Apparatus

All analyses were performed on an HPCE apparatus (CL1030, Beijing Cailu Instrumental Co., Beijing, China), equipped with a power supply, a HW-2000 chromatography workstation and a UV-visible detector. An uncoated fused-silica capillary column (Hebei Optical Fiber, China) with a total length of 49 cm (effective length 40 cm to detector) × 50 μm i.d. was used for separations throughout the experiments.

A new capillary was conditioned by flushing with 1.0 mol/L NaOH for 1 h and water for 30 min. At the beginning of each working day, the capillary was rinsed successively with 0.1mol/L NaOH for 5 min, water for 5 min, and running BGE for 10 min. Between each injection, it was purged with running BGE for 2 min. The detection wavelength was set at 271 nm for levo-folinic acid and (6*R*,2'*S*)-diastereomer. An applied voltage from 15 to 30 kV was used for optimization of the resolution during the separation. Samples were injected into the capillary by hydrodynamic flow at a height differential of 10 cm for 5 s. Every sample solution was injected two times for each experimental condition. Acetone was used as a neutral marker to determine the electroosmotic flow (EOF).

The BGEs were prepared by dissolving the CD in an appropriate concentration of NaH_2_PO_4_ buffer and the desired pH adjustment was carried out with 1.0 mol/L H_3_PO_4_ or 0.1 mol/L NaOH. Sample stock solutions were prepared in methanol at 1.0 mg/mL and were stored in a refrigerator. The stock solutions were further diluted ten times with double distilled water to give sample solutions. Filters with a pore size of 0.25 μm were used to filter all the solutions.

## Results and Discussion

### Effect of BGE pH

The effect of buffer pH on diastereoseparation of the studied analytes was investigated over the range of 2.75 to 7.00 using 30mM phosphate buffer with 6.0 mmol/L PIP-*β*-CD (Table 1). Under such conditions, PIP-*β*-CD was completely protonated (pKa ~11 from the piperidine group). In the case of folinic acid (H_2_A, pKa_1_ = 3.1 and pKa_2_ = 4.8), it has different degrees of dissociation at various pH. Therefore, the effect of pH on the migration time (*t*) and resolution (*R*_s_) of the folinic acid diastereomers is more complicated due to the fact that the analyte possesses two ionizable carboxylate in its structure.

**Table 1:** The effect of the pH on time of EOF (*t*_EOF_), the migration time (*t*), resolution (*R*_s_) and separation selectivity (*α*) of folinic acid diastereomers.

pH	*t*_EOF_ (min)	*t*_1_ (min)	*t*_2_ (min)	*R*_s_	*α*
2.75	21.63	20.91	21.00	0.98	1.004
3.00	21.08	24.22	26.03	1.84	1.075
3.25	20.82	26.35	27.68	2.03	1.051
6.00	6.20	29.39	30.64	1.91	1.042
6.25	5.70	26.00	27.05	2.10	1.040
6.50	5.31	23.56	24.42	2.31	1.037
6.75	4.38	18.43	18.89	1.48	1.025
7.00	3.91	15.44	15.75	0.91	1.020

Note: In pH range of 3.50–5.75, no peaks of folinic acid diastereomers were detected within 60 min. Each data is the mean of two injections.

The enantioseparation parameters of CE were calculated as follows: *R*_s_ = 2(*t*_2_-*t*_1_) / (*W*_2_+*W*_1_); *α* = *t*_2_/*t*_1_; where *t*_1_ and *t*_2_ are the migration times of isomer 1 and 2 and *W*_1_ and *W*_2_ are the peak widths at baseline of each isomer. 1: (6*R*,2'*S*)-diastereomer 2: levo-folinic acid

BGE: 6.0 mmol/L PIP-*β*-CD, 30 mmol/L phosphate buffer containing 10% acetonitrile, 20 kV

At pH 2.75, the analytes were less dissociated, and the complexation between the positively charged CD and folinic acid gave the latter a pseudopositive charge. Therefore, this complex moved towards the cathode. With the pH increased to 3.25, the dissociation (from H_2_A to HA^-^) of analytes was increased. As a result, they acquired negative charge resulting in their negative electrophoretic mobilities increased rapidly, and tended to move toward the anode. As observed, the resolution and migration times of folinic acid diastereomers were increased, and a *R*_s_ of 2.03 was obtained at pH 3.25. It can be concluded that both the longer time and the more electrostatic interactions between CD and analytes contributed to the separation.

When pH was continuously increased from 3.25 to 5.75, folinic acid diastereomers gradually dissociated (form HA^-^ to HA^2-^), so that the negative electrophoretic mobility of the analytes were increased rapidly and were even larger than EOF. Due to the fact that the net migration of the analytes was so small as to be detected, no peak was observed in 60min.

Subsequently, pH values were gradually increased from 6.00 to 7.00, and in this pH range the EOF became the dominant factor affecting migration of the diastereomers. As pH increased, the EOF increased rapidly, so that the migration times of analytes were shortened. Apart from EOF, the change of protonation degree of carboxyl groups in folinic acid also influenced the separation process in this pH range. The mobilities of folinic acid anionic form were increased due to the excessive ionization of carboxyl groups (from HA^-^ to A^2-^). The increase in negative charge of analytes also increases the electrostatic attraction, which in turn increases diastereoseparation. However, increasing electroosmotic mobility caused the migration time of the analytes to decrease, offering less opportunities for analytes-CD interactions. As a result, the maximum *R*_s_ of 2.31 was obtained at pH 6.50. Generally, the max separation selectivity (α) was accompanied with the max *R*_s_. In our study, at pH 6.50, the *α* value was lower than that at pH 3.25, but with a higher resolution. The explanation might be that at acidic pH the analytes exist in two forms (H_2_A and HA^-^), resulting in wide peaks of the analytes and a decrease of resolution. Therefore, considering both good resolution and proper migration time, pH 6.50 was proved to be the optimum BGE pH to separate the diastereomers and was used in the further investigation.

### Effect of organic modifier

The addition of organic modifiers to the BGE can alter several parameters, such as the polarity and/or viscosity of BGE, and the EOF, which could influence the electrophoresis migration behaviors of the analytes, such as the migration time and resolution.

Herein, two organic additives, methanol and acetonitrile with a percentage content of 0%, 2%, 5% and 10% were investigated in BGE with 30 mmol/L phosphate buffer containing 6.0 mmol/L PIP-*β*-CD at pH 6.50. Interestingly when the organic solvents were present in the BGE, the selectivity, resolution and migration time did not change markedly for the pairs of diastereomers under these conditions. However, the peak shape of the pairs of folinic acid diastereomers was markedly improved when 10% acetonitrile was added. Therefore, 10% acetonitrile was used in the BGE.

### Effect of PIP-*β*-CD concentration

The investigation of PIP-*β*-CD concentration on separation performance was performed in pH 6.50 BGE containing 10% acetonitrile. The migration time of EOF (*t*_EOF_), the *t*, effective mobility (*μ*_eff_), defference of *μ*_eff_ (Δ*μ*_eff_), *R*_s_ and *α* of folinic acid diastereomers under varied CD concentration are summarized in Table 2. From Table 2 it could be seen that increase in the concentration of PIP-*β*-CD from 0 to 7.5mmol/L generally led to a decrease in EOF as a result of elevated viscosity and ionic strength of buffer with addition of selector. Also, the *μ*_eff_ values of studied analytes were dependent on CD concentration. The increase of PIP-*β*-CD concentration generally led to decreased of *μ*_eff_ (sign"-" indicating the self migration of analyte was in the opposite direction of EOF), resulting from that the interaction between analytes and the CD was strengthened.

**Table 2:** The effect of the concentration of PIP-*β*-CD on time of EOF (*t*_EOF_), the migration time (*t*), effective mobility (*μ*_eff_), defference of *μ*_eff_ (Δ*μ*_eff_), resolution (*R*_s_) and separation selectivity (*α*) of folinic acid diastereomers.

C (mmol/L)	*t*_EOF_ (min)	*t*_1_ (min)	*t*_2_ (min)	*μ*_eff1_ (×10^-8^m^2^/Vs)	*μ*_eff2_ (×10^-8^m^2^/Vs)	Δ*μ*_eff_	*R*_s_	*α*
0.0	3.90	10.80	10.80	-2.678	-2.678	0.0	0.00	1.000
1.5	4.57	16.16	16.33	-2.567	-2.577	-1.05	0.79	1.010
3.0	4.76	18.03	18.40	-2.527	-2.545	-1.81	1.28	1.020
4.5	5.07	20.81	21.40	-2.438	-2.460	-2.18	1.70	1.029
6.0	5.31	23.56	24.42	-2.386	-2.410	-2.45	2.31	1.037
7.5	5.63	25.29	26.05	-2.255	-2.274	-1.89	2.00	1.030

Note: Each data is the mean of two injections. The enantioseparation parameters of CE were calculated as follows: *μ*_eff_ = (1/*t-*/*t*_EOF_)lL/V; Δ*μ*_eff_ = *μ*_eff2_-*μ*_eff1_; where *t*_1_ and *t*_2_ are the migration times of isomer 1 and 2. 1: (6*R*,2'*S*)-diastereomer 2: levo-folinic acid

BGE: pH 6.50, 30 mmol/L phosphate buffer containing 10% acetonitrile, 20 kV

**Fig 2:**
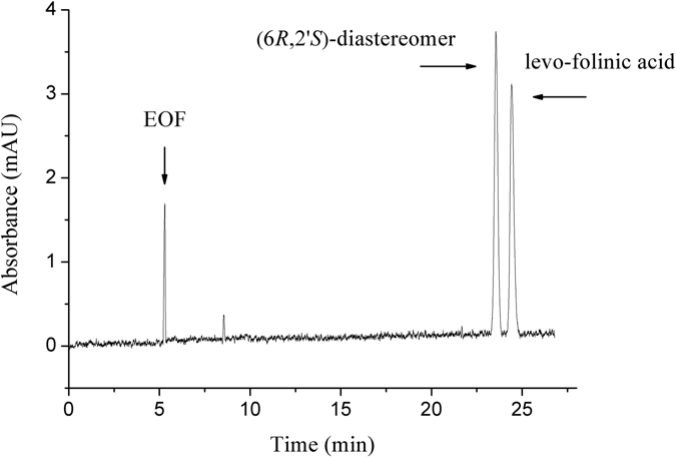
The optimum electropherogram of separation of folinic acid diastereomers. BGE: pH 6.50, 6.0 mmol/L PIP-*β*-CD, 30 mmol/L phosphate buffer containing 10% acetonitrile, 20 kV.

The effect of PIP-*β*-CD concentration on *R*_s_ showed more or less a similar trend as that on *α*. They first increased with the increment in selector concentration then continuously decreased. The Maximum *R*_s_ and *α* values were observed at PIP-*β*-CD of 6.0 mmol/L corresponding to their max Δ*μ*_eff_ value. Accordingly, 6.0 mmol/L PIP-*β*-CD was selected for the final method and was found to provide a good resolution and a suitable run time. Fig. 2 showed the optimum electropherogram for the separation of the folinic acid diastereomers. According to the theoretic model developed by Wren and Rowe [14, 15], the CD concentration corresponding to the maximum apparent mobility difference (Δ*μ*_eff_) should give the best chiral resolution, which was confirmed by the resolution data in Table 2.

The precision of our proposed method is expressed by relative standard deviation (RSD). The RSD value was evaluated within-day (n = 6) and between-day (n = 6, day = 3) based on the folinic acid diastereomers separation with 30 mmol/L phosphate buffer, containing 6.0 mmol/L PIP-*β*-CD at pH 6.50 with 10% ACN. The RSD values of migration time for the levo-folinic acid and its (6*R*,2'*S*)-diastereomer were 1.2% and 1.4% for within-day, 2.2% and 2.3 for between-day, respectively. Additionally, the RSD value of Rs was 1.1%. Based on the results, this newly synthesized mono-substituted cationic PIP-*β*-CD showed well precision and repeatability using in CE.

### Determination of binding constants

The binding constants were calculated from the measured mobility values of each diastereomer in pH 6.50 BGE containing 0–7.5 mmol/L selector. In this study, assuming 1:1 stoichiometry for the complex forming between CD selector and selected, the theoretical model relating the mobility to the concentration of the CD selector developed by Wren and Rowe [14,15] was used, 1/(*μ*_f_-*μ*_eff_) = 1/(*μ*_f_-*μ*_c_)*K*[CD]+1/(*μ*_f_-*μ*_c_), where *K* is the binding constant, [CD] is the total CD concentration, *μ*_eff_ is the effective electrophoretic mobility observed for ether isomer, and *μ*_f_ and *μ*_c_ are the electrophoretic mobilities of the isomers in the free and complexed forms, respectively. Under the experimental conditions, changes in the viscosity of the BGE when concentration of CD increases affect the mobility of the analytes, and this may affect the results. Godall and co-workers [16] suggested a viscosity correction based on the measurement of the current during CE experiments. Values of current in the absence and presence of CD (*I*_0_ and *I*) were used to rectify the viscosity effect: *μ*_eff_ values were corrected by the ratio *I*_0_/*I*.

If the variations of 1/(*μ*_f_-*μ*_eff_) vs. 1/[CD] are straight lines (*R* ^2^>0.99), *K* can be extracted from the slope and intercept. The binding constants *K* of levo-folinic acid and its (6*R*,2'*S*)-diastereomer was 154.96 L/mol and 147.48 L/mol, respectively, the and *C*_opt_ calculated from *K* was 6.6 mmol/L. It is noteworthy that the optimum selector concentration calculated according to the model was very close to the experiment one giving the maximum resolution of the folinic acid diastereomers. Based on the above analysis, it is very obvious that PIP-*β*-CD have a significant *K* value towards folinic acid diastereomers. Meanwhile, the (6*R*,2'*S*)-diastereomer, having a higher apparent complex stability constant with PIP-*β*-CD than levo-folinic acid, was eluted first, which was consistent with the experimental results.

### Computational modeling

To further understand how diastereomers separation takes place at the atomic level, we used computer modeling techniques to investigate the interaction between PIP-*β*-CD and folinic acid diastereomers. All theoretical calculations were performed using Gaussian 09 software packages. The initial structures of two folinic acid diastereomers obtained from ChemBioDraw Ultra 12.0 were optimized by using Gaussian 09 (Gaussian, Wallingford, CT, USA), at the density functional theory level with a B3LYP functional and 6–31G(d, p) basis set. And the initial structure of PIP-*β*-CD was optimized by semi-empirical PM3 method.

**Fig 3:**
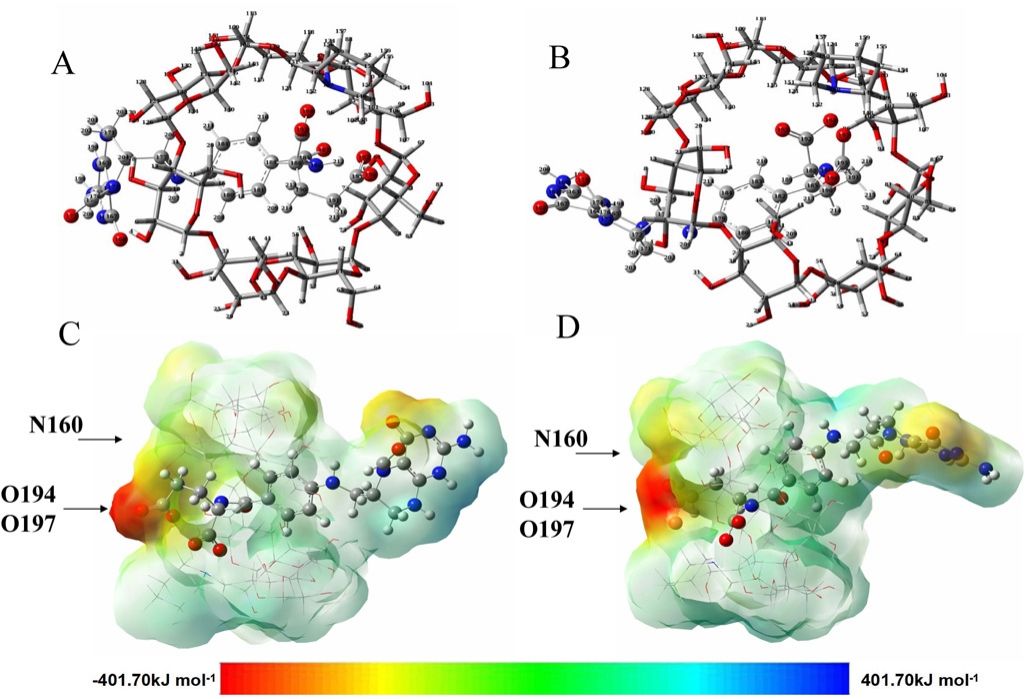
The optimized geometries for the lowest energy conformation for the inclusion complexes of folinic acid diastereomers with PIP-*β*-CD. (A) levo-folinic acid with PIP-*β*-CD. (B) (6*R*,2'*S*)-diastereomer with PIP-*β*-CD. The molecular electrostatic potential (MEP) maps of the optimized conformation for the inclusion complexes of folinic acid diastereomers with PIP-*β*-CD. (C) levo-folinic acid with PIP-*β*-CD. (D) (6*R*,2'*S*)-diastereomer with PIP-*β*-CD.

Firstly, using the optimized structures above, the semi-empirical PM3 method [12] was applied to search for the most stable inclusion complexation of folinic acid diastereomers into PIP-*β*-CD cavity from its wide (Model I) and narrow (Model II) sides by scanning the coordinate Z, from -8A to 8 A intervals. The calculated binding energies (Δ*E*) associated with the formation complexes of folinic acid diastereomer with PIP-*β*-CD are obtained using following equation: Δ*E* = *E*_complex_ − (*E*_free-guest_ + *E*_free-host_), where *E*_complex_, *E*_free-guest_, *E*_free-host_ are the energies of complexes of folinic acid diastereomer with PIP-*β*-CD, free folinic acid diastereomer and free PIP-*β*-CD, respectively. The difference of Δ*E* of each isomer with PIP-*β*-CD is calculated using following equation: ΔΔ*E* = Δ*E*_levo-folinic acid /(PIP-*β*-CD)_ − Δ*E*_(6*R*,2'*S*)-diastereomer /(PIP-*β*-CD)_, where Δ*E*_levo-folinic acid /(PIP-*β*-CD)_ means the calculated binding energies of complexes of levo-folinic acid with PIP-*β*-CD, and Δ*E*_(6*R*,2'*S*)-diastereomer /(PIP-*β*-CD)_ means the calculated binding energies of complexes of (6*R*,2'*S*)-diastereomer with PIP-*β*-CD. For model I, Δ*E*_levo-folinic acid /(PIP-*β*-CD)_ was -35.79 kJ/mol, Δ*E*_(6*R*,2'*S*)-diastereomer /(PIP-*β*-CD)_ was -46.15, and ΔΔ*E* was 10.36 kJ/mol. For model II, ΔE _levo-folinic acid /(P-*β*-CD)_ was -31.98 kJ/mol, Δ*E*_(6*R*,2'*S*)-diastereomer /(PIP-*β*-CD)_ was -42.02, and ΔΔ*E* was 10.04 kJ/mol. On account of the principle that the more negative the binding energy is, the stronger interaction takes place between the host and guest molecules and the more stable is the corresponding host-guest complex. Therefore, compared with the wide and narrow sides by which guest molecules entered from the cavity, it conforms that the complexation energies are in favor of model I. According to the energy values of each stereoisomers cyclodextrin complex, the order of stability of two folinic acid diastereomers was (6*R*,2'*S*)-diastereomer > levo-folinic acid indicating (6*R*,2'*S*)-diastereomer had a stronger interation with PIP-*β*-CD, which was consistent with the peak order in the previous experiments (Fig. 2). The optimized geometries for the lowest energy conformation for the inclusion complexes of two folinic acid diastereomers with PIP-*β*-CD are presented in Fig. 3A and 3B.

Secondly, for the further purpose of understanding the information about the region from where and how the PIP-*β*-CD and the analytes can have intermolecular interactions to form the above optimized inclusion complexes, the molecular electrostatic potential was investigated by using the PM3 method. The molecular electrostatic potential (MEP) is a useful feature that it simultaneously displays molecular size, shape as well as positive, negative and neutral electrostatic potential regions in terms of color grading and is very useful in research of molecular structure with its physiochemical property relationship. In the MEP maps, the different values of the electrostatic potential at the surface are represented by different colours, for example, red color stands for the maximum negative region and blue color stands for the maximum positive region, and potential increases in the order red<orange<yellow<green<blue. As can be seen from the Fig. 3C and 3D, the O194 and O197 atoms in carbonyl groups of folinic acid diastereomers were surrounded by negative charge, making these sites more favourable for possible coordination points. The maximum values of the negative region on the O194 and O197 atoms in carbonyl groups of levo-folinic acid were determined as -119.73 and -291.45 kcal/mol, and a maximum positive region localized on the N160 atom of the piperidine ring had a value of 52.18 kcal/mol, indicating a possible site for molecular electrostatic attack interaction. For diastereomer, the maximum values of negative region on the O194 and O197 were -125.10 and -300.86 kcal/mol, and the maximum value of positive region was 56.87 kcal/mol. These larger values further confirmed that the (6*R*,2'*S*)-diastereomer had a stronger interaction with PIP-*β*-CD. Considering the calculated results and also the MEP maps, these gave the information about the region from where and how the analytes can have intermolecular electrostatic interactions with the PIP-*β*-CD, which might be a dominant and helpful feature for the understanding the mechanism occurrence of the separation of anionic folinic acid diastereomers by cationic PIP-*β*-CD.

## Conclusions

In this paper, a newly synthesized cationic single-isomer CD derivative, PIP-*β*-CD, was utilized as a selector to separate folinic acid diastereomers in CE. The effect of various parameters like BGE pH, PIP-*β*-CD concentration, and nature and concentration of organic modifier was studied during the method optimization process. At a low PIP-*β*-CD concentration of 6.5 mmol/L, a good separation of diastereomers of folinic acid with *R*_s_ of 2.31 was obtained. This experimental concentration was quite close to the optimum *C*_opt_ calculated from the determined binding constants. Furthermore, a computational modeling strategy was used in order to discuss the possible separation mechanism, and according the calculated binding energies, the most stable host-guest complexes was formed when the folinic acid diastereomers moved into PIP-*β*-CD cavity from the wide side. Based on these stable complexes, the MEP maps were obtained and showed that the intermolecular electrostatic interactions between the analytes and CD happened to be the most important factor influencing the separation. In conclusion, PIP-*β*-CD displayed effective stereoselective separation ability towards the selected analytes, which could favorably broaden the scope of CE enantioseparation with single-isomer cationic CDs as selectors.

## Supporting Information

S1 Table Effect of organic modifier. The effect of organic modifier on enantioseparation parameters of (6*R*,2'*S*)-diastereomer (the first peak).
(DOC)
