# Supplementary material for: Separation of Folinic Acid Diastereomers in Capillary Electrophoresis Using a New Cationic β-Cyclodextrin Derivative
Source: PLoS One. 2015 Mar 17;10(3):e0120216. doi: 10.1371/journal.pone.0120216 (PMC4364531; doi:10.1371/journal.pone.0120216)
Supplement: S1 Table — The effect of organic modifier on enantioseparation parameters of (6R,2'S)-diastereomer (the first peak). (DOC) [file pone.0120216.s001.doc]

**S1_Table. The effect of organic modifier on enantioseparation parameters of (6*R*,2'*S*)-diastereomer** (the first peak).

|  | *t* (min) | *R*_s_ | *W_1/2_*(s) | *N* |
| --- | --- | --- | --- | --- |
| No organic modifier | 25.90 | 2.10 | 14.30 | 65408 |
| 2% methanol | 25.89 | 2.00 | 14.56 | 63114 |
| 5% methanol | 25.40 | 1.96 | 14.72 | 59426 |
| 10% methanol | 24.82 | 1.86 | 15.52 | 51023 |
| 2% acetonitrile | 25.70 | 2.10 | 14.29 | 64519 |
| 5% acetonitrile | 24.45 | 2.12 | 13.13 | 69136 |
| 10% acetonitrile | 23.56 | 2.31 | 12.54 | 70371 |

**Note:** Each data is the mean of two injections.

The enantioseparation parameters of CE were calculated as follows:

*N*= 5.54(*t*_1_/*W*_1/2_ _(1)_)^2^, where *t*_1_ is the migration time of (6*R*,2'*S*)-diastereomer, *W*_1/2(1)_ is the half-peak width of (6*R*,2'*S*)-diastereomer.

BGE: pH 6.50, 6.0 mmol/L PIP-*β*-CD, 30 mmol/L phosphate buffer, 20 kV
